# Working Memory and Attention – A Conceptual Analysis and Review

**DOI:** 10.5334/joc.58

**Published:** 2019-08-08

**Authors:** Klaus Oberauer

**Affiliations:** 1 University of Zurich, CH

**Keywords:** Working memory, Attention, Cognitive Control

## Abstract

There is broad agreement that working memory is closely related to attention. This article delineates several theoretical options for conceptualizing this link, and evaluates their viability in light of their theoretical implications and the empirical support they received. A first divide exists between the concept of attention as a limited resource, and the concept of attention as selective information processing. Theories conceptualizing attention as a resource assume that this resource is responsible for the limited capacity of working memory. Three versions of this idea have been proposed: Attention as a resource for storage and processing, a shared resource for perceptual attention and memory maintenance, and a resource for the control of attention. The first of these three is empirically well supported, but the other two are not. By contrast, when attention is understood as a selection mechanism, it is usually not invoked to explain the capacity limit of working memory – rather, researchers ask how different forms of attention interact with working memory, in two areas. The first pertains to attentional selection of the contents of working memory, controlled by mechanisms of filtering out irrelevant stimuli, and removing no-longer relevant representations from working memory. Within working memory contents, a single item is often selected into the focus of attention for processing. The second area pertains to the role of working memory in cognitive control. Working memory contributes to controlling perceptual attention – by holding templates for targets of perceptual selection – and controlling action – by holding task sets to implement our current goals.

There is a broad consensus that working memory and attention are intimately linked (Awh, Jonides, & Reuter-Lorenz, 1998; Baddeley, 1993; Chun, 2011; Cowan, 1995; Gazzaley & Nobre, 2012; Kane, Bleckley, Conway, & Engle, 2001; Kiyonaga & Egner, 2014; Oberauer, 2009; Olivers, 2008). But what is it that we agree upon? Both working memory and attention can be conceptualized in different ways, resulting in a broad array of theoretical options for linking them. The purpose of this review is to propose a map for organizing these theoretical options, delineate their implications, and to evaluate the evidence for each of them.

The meaning of the concept *working memory* (WM) depends on the theory in which the concept figures. The definitions reviewed by Cowan (2017) differ primarily in the substantive assumptions they include (e.g., whether or not WM consists of multiple storage modules, and to what extent it includes long-term memory). Beyond these differences in theoretical assumptions, however, there is a broad consensus on what the term *working memory* refers to: The mechanisms and processes that hold the mental representations currently most needed for an ongoing cognitive task available for processing.

The meanings of the term *attention* are more diverse, as they reflect distinctions not only of definitions but also of different referents of the term: Attention is not a unitary entity (Chun, Golomb, & Turk-Browne, 2011). Conceptualizations of attention can be distinguished along several dimensions that provide a coordinate system for our conceptual map. A first distinction pertains to how *attention* is defined. One definition of attention characterizes it as a limited resource for information processing (e.g., Wickens, 1980). Another concept of attention is as a process of (or mechanism for) selection of information to be processed with priority (e.g., Chun et al., 2011; Desimone & Duncan, 1995). These two concepts of attention play different roles in theorizing about working memory, and I will discuss them in turn below.

A second distinction pertains to what we attend to. I find it useful to distinguish the possible objects of attention along two dimensions (see Table 1).^1^ First, we can distinguish between attention to our currently perceived environment (e.g., attention to visual objects or auditory streams) from attention to information currently not perceived, such as attention to remembered episodes or concepts that we think about.^2^ Second, we can distinguish between attention to things and events in the world around us on the one hand, and attention to our own goals and (mental or overt) actions on the other. The latter form of attention includes selection of our current goal or task set and shielding it from distraction (Kane & Engle, 2003; Monsell, 2003), selection of one of several possible actions (Pashler, 1994), and monitoring of our actions and their outcomes (Yeung, Botvinick, & Cohen, 2004).

**Table 1:** A Taxonomy of Attention.

	Controlled *Limited resource for controlling attention*		Automatic *Not resource limited*	

	Things/Events	Goals/Actions	Things/Events	Goals/Actions

Perceptual	Selective attention to locations, visual objects, events, features. *Limited capacity for attending several channels.*	Selective attention to ongoing actions, monitoring of action outcomes	Capture of attention by salient stimuli, to stimuli learned to be relevant, or to stimuli in the focus of attention of WM	Capture of attention by errors or unexpected difficulties.
Non-Perceptual	Attention to items in WM. *Limited capacity for maintenance (“storage”)*	Attention to intended actions: Selection of task sets, response selection. *Central processing capacity*	Involuntary retrieval from long-term memory; intrusive thoughts.	Involuntary retrieval of task sets associated to current stimulus; involuntary selection of response (e.g., Stroop, flanker task)

*Note:* Descriptions pertaining to attention as selection/prioritization are printed in regular font; descriptions pertaining to attention as a resource in italics.

A third distinction pertains to the forces that determine what we attend to – this is the distinction between controlled and automatic deployment of attention (Shiffrin & Schneider, 1977). Attention is controlled when it is directed according to our current goals. The influence of current goals on attention is often referred to as “top-down”. Attention is automatic to the extent that its direction is influenced by forces independent of our current goals – these include the “bottom-up” attraction of attention by perceived properties of the stimuli (e.g., their “salience”) as well as influences of our learning history on what we attend to, for instance when attention is drawn to information that we have learned to be relevant (Awh, Belopolsky, & Theeuwes, 2012; Theeuwes, 2018).

The concept of *executive attention* is often used when discussing the relation between attention and working memory. *Executive attention* is a term that is notoriously poorly defined (Jurado & Rosselli, 2007). It is used on the one hand to refer to attention directed to one’s own goals and (mental or overt) actions, including response selection (Szmalec, Vandierendonck, & Kemps, 2005), action planning, protecting the pursuit of our current goal from distractions and temptations, as well as switching from one task to another. On the other hand, executive attention is also used to refer to the top-down control of attention, including attention to things and events in the environment – for keeping our attention on the relevant stimuli or features and avoiding distraction by irrelevant ones, as in the Stroop task and the flanker task. As such, the term *executive attention* is used to denote one pole on each of two dimensions in my proposed taxonomy, one pertaining to the objects of attention (things and events in the world vs. our own goals and actions), the other pertaining to what determines the orientation of attention (controlled vs. automatic). The first meaning assigns executive attention a function in controlling our thoughts and actions (including what we attend to) whereas the second states that executive attention is itself controlled. One way to perhaps bring together the two meanings is by assuming that we attend to (i.e., select, assign resources to) our own goals and actions – including the action of attending to some object – in order to control them. Nevertheless, I find the term *executive attention* disquietingly ambiguous, and therefore will use instead the terms *attention to (cognitive) action* and *controlled attention* to refer to the two aspects of executive attention, respectively.

I organize the review by the two definitions of attention – as a resource or as a selection mechanism – because they have different implications for how attention and working memory are related. Within each section I will discuss the different objects of attention, and the different modes of control.

## Attention as a Resource

The idea of attention as a resource is that the cognitive system has a limited resource that can be used for carrying out so-called attention-demanding processes. The resource is assumed to be a continuous quantity that can be split arbitrarily and allotted to different processes, depending on task demands. Processing efficiency (i.e., speed, accuracy) is a positive monotonic function of the amount of resource assigned to a process (Navon & Gopher, 1979). The assumption that WM capacity reflects a limited resource has a long tradition (Anderson, Reder, & Lebiere, 1996; Case, 1972; Just & Carpenter, 1980; Ma, Husain, & Bays, 2014). Authors linking WM to an attentional resource are endorsing the view that the limited capacity of WM reflects a limited resource, and that this resource also serves some (or all) functions commonly ascribed to attention. Three versions of this idea can be distinguished by which functions the attentional resource is assumed to be needed for: (1) storage and processing of information (e.g., Just & Carpenter, 1992), (2) perceptual attention and memory maintenance (e.g., Ester, Fukuda, May, Vogel, & Awh, 2014; Kiyonaga & Egner, 2014), or (3) the control of attention (e.g., Allen, Baddeley, & Hitch, 2006; Baddeley, 1993, 1996; Lavie, 2005).

### Attention for Storage and Processing

Many theorists discussing the relation between working memory and attention characterize attention as a limited resource for maintaining representations in an “active”, available state (Cowan, 2005). Often this resource is assumed to be shared between “storage” and “processing” (Case, Kurland, & Goldberg, 1982; Cowan et al., 2005; Just & Carpenter, 1992). According to this view, the same attentional resource is required for keeping representations available and for carrying out certain basic cognitive processes such as selecting a response to a stimulus. A prediction from this theory is that attention-demanding cognitive processes compete with concurrent storage (Z. Chen & Cowan, 2009).

There are two variants of this theoretical idea. One is that a share of the resource needs to be continuously assigned to a representation to keep it in WM (Case et al., 1982). The other is that attention is required directly only for processing, not storage. In this view attention indirectly contributes to memory maintenance because it is needed for refreshing WM representations, which would otherwise decay (Barrouillet, Bernardin, & Camos, 2004). Barrouillet and colleagues further specify the resource required for refreshing as the limited resource for so-called central processes, such as response selection (Barrouillet, Bernardin, Portrat, Vergauwe, & Camos, 2007). Dual-task studies with variants of the PRP (psychological refractory period) paradigm have established a strong capacity limit on central processes (Pashler, 1994), which has been explained by a limited central-attentional resource (Navon & Miller, 2002; Tombu & Jolicoeur, 2003).

Theorists linking WM to attention as resource commonly assume that there is a single, content-general attentional resource. It follows that storage and processing compete with each other whether or not they share any contents. This assumption leads to the prediction of dual-task costs when WM storage and processing demands from very different contents are combined with each other. There is considerable evidence confirming this prediction (Chein, Moore, & Conway, 2011; Morey & Bieler, 2012; Saults & Cowan, 2007; Vergauwe, Barrouillet, & Camos, 2010), lending support to the notion that WM capacity is limited by an attentional resource. There is also evidence that storage and processing compete for central processing capacity: The extent to which maintenance in WM is impaired by concurrent processing is a monotonic function of *cognitive load*, defined as the proportion of time during which central attention is engaged by the processing demand (Barrouillet et al., 2007).

One problem for the assumption of a shared resource for storage and processing is that, although a memory load reduces the efficiency of concurrent response-selection tasks, that dual-task cost diminishes substantially over the first few seconds of the retention interval (Jolicoeur & Dell’Acqua, 1998; Thalmann, Souza, & Oberauer, 2019; Vergauwe, Camos, & Barrouillet, 2014), and is often not observed at all when there is an unfilled interval of a few seconds between encoding of the memory set and commencement of the processing task (Hazeltine & Witfall, 2011; Klapp, Marshburn, & Lester, 1983; Oberauer, Demmrich, Mayr, & Kliegl, 2001). This observation has already led Klapp and colleagues (1983) to question the idea of a shared resource for storage and processing: To uphold this idea we would have to assume that the resource demand of maintenance dwindles to a negligible level within a few seconds. This would be compatible with the assumption that a central processing resource is required for short-term consolidation of information in working memory (Jolicoeur & Dell’Acqua, 1998; Nieuwenstein & Wyble, 2014; Ricker & Hardman, 2017) but not with the assumption that a resource is needed for maintenance throughout the retention interval.

As mentioned above, the assumption of shared resources for storage and processing comes in two variants: The first, traditional one is that a representation needs a share of the resource assigned to it to be in WM, and the same resource is needed for carrying out cognitive operations. The second variant is that maintenance processing such as refreshing share a limited resource with other cognitive operations (Barrouillet et al., 2004). The second variant rests on the premise that without refreshing the representations in WM decay – only on that assumption does the processing resource assigned to refreshing become essential for WM maintenance. The decay assumption, however, is probably not true, at least for verbal materials (Oberauer & Lewandowsky, 2013, 2014).

The first variant has a conceptual problem: Simultaneous maintenance and processing compete for a shared resource only until the processing task is completed – after that, the full resource can be re-assigned to the representations in WM. Why then should memory performance suffer from a concurrent processing task although memory is tested only after the processing task is done? (for a more detailed treatment see Oberauer, Farrell, Jarrold, & Lewandowsky, 2016). The problem is illustrated by a study that, according to the authors, reveals the neuronal basis of resource sharing: Watanabe and Funahashi (2014) recorded from multiple neurons in the lateral pre-frontal cortex (LPFC) while monkeys did a spatial attention task, a spatial WM task, or a dual-task combination of the two. The two tasks recruited largely overlapping LPFC neurons, which showed spatial selectivity when each task was done alone. While both tasks were done simultaneously, the LPFC neurons lost most of their spatial selectivity, and collectively their firing rate pattern contained less information about the attended location and the remembered location during that period. After the attention task was completed, however, the information about the location in memory was “reawakened” in the firing pattern of the LPFC neurons, reaching the same strength as in the single-task condition. The authors did observe a (small) performance decrement in the dual-task relative to the single-task condition, but that dual-task cost is not explained by their neural data – looking at the neural data, we would expect no detrimental effect on memory by the concurrent attention task.

To conclude, the assumption of a shared resource for memory retention and central processes has received much empirical support. At the same time, it is challenged by the finding that dual-task costs on processing speed tend to vanish over time, and – depending on the version endorsed – the lack of evidence for decay, and the problem of how to explain that the competition between processing and storage affects memory performance after the competition has ended.

### Attention for Perception and Memory

A resource shared between “storage” and “processing” spans both sides of the distinction between attention to things and events (i.e., the information to be stored), and attention to goals and actions (i.e., to the task sets guiding the processing operations). We can also ask whether the same resource applies to both sides of another distinction, the one between perceptual attention and attention to not-perceived objects. Most task paradigms for studying WM require retention of information in the absence of perceptual input. There is evidence, however, that the limited capacity of WM applies not only to information in memory but equally to information still in view. Tsubomi, Fukuda, Watanabe, and Vogel (2013) measured the contralateral delay activity (CDA), a neural marker of the number of objects a person holds in visual WM (Luria, Balaban, Awh, & Vogel, 2016; Vogel & Machizawa, 2004) while participants attended to a variable number of color patches still in view, or attempted to remember them after their offset. In both cases, the CDA amplitude increased with set size up to about 3 items and then levelled off. Individual CDA amplitudes correlated with performance on a test of one randomly selected item regardless of whether that item remained in view until the time of test or had to be retained in memory for a second.

The study of Tsubomi et al. (2013) shows striking similarities between the capacity limits for attending to perceptual stimuli and for maintaining stimuli in memory (see also Ester et al., 2014). Still, these two functions could rely on separate resources that happen to bear similarities to each other. If the same limited resource underlies perceptual attention and maintenance in WM, then demanding both at the same time should incur a substantial dual-task cost, such that when the load of one task is increased, performance on the other suffers. The evidence for this prediction is mixed. Fougnie and Marois (2006) found load-dependent dual-task costs when combining a visual WM task with a visual attention task (simultaneous tracking of multiple moving objects, or monitoring multiple parallel streams of rapidly presented visual stimuli for a target) but these costs were less than the cost of combining two visual WM tasks. Souza and Oberauer (2017) found only negligible dual-task costs when inserting a visual attention task (monitoring a stimulus for a subtle brightness change) in the retention interval of a visual WM task. Several studies investigated dual-task costs between WM and visual search. These dual-task costs increase with the load on each of the two tasks – as expected on the assumption of a shared resource – only when the contents of WM were spatial locations (for a review see Woodman & Chun, 2006). To conclude, although attending to perceptual information and maintaining information in WM after it disappeared from the environment have much in common, the evidence that they share a limited resource is not yet convincing.

### Controlled Attention

The concept of attention as a limited resource is often linked specifically to controlled attention, whereas automatic attention is thought not to be resource demanding (Schneider & Shiffrin, 1977; Shiffrin & Schneider, 1977). There are two ways in which this link can be spelled out: (a) Attention that is allocated in a controlled manner – according to “top down” influences from our current goals – underlies a resource limit but attention that is automatically attracted to some information independent of its relevance for our current goal does not underlie that resource limit. Stated in this way we face the awkward conclusion that allocating attention to the same object (e.g., a red traffic light in a street scene, or a word we hold in WM) does or does not rely on a limited resource depending on what forces led attention to that object. The same cognitive function – prioritizing processing of the attended information – would be resource consuming or not depending on how it was invoked.

In my view, a less awkward interpretation is: (b) Paying attention to an object does not require a resource per se – rather the process of controlling attention in a top-down manner consumes the limited resource. This interpretation reflects how Shiffrin and Schneider (1977, p. 156) explain why controlled processes are capacity limited: These processes need to be controlled by continuously paying attention to them, and attention cannot be allocated to more than one process at a time. In other words, the attentional resource imposes a bottleneck on the *control* processes, not on the *controlled* processes. The limitation is on how many different (cognitive or overt) actions we can attend to at the same time in order to control them. For instance, in visual search, perceptual attention can be drawn to some stimuli automatically, and theoretically there is no limit on how many such forces exert their pull in parallel. Perceptual attention can also be directed in a controlled manner – by attending to the action of deploying attention to visual stimuli – and this control process is limited to one action at a time. The limitation does not rest with the controlled attention – a limit on how many visual stimuli can be attended at the same time – but with the controlling attention.

This conception of an attentional resource differs from the preceding two. The notion of a resource for storage and processing and the idea of a shared attentional resource for perception and memory share the assumption that the resource is allocated to representations of objects and events that we perceive or hold in WM. In contrast, the “attentional control” idea assumes a resource for the control of what we attend to, and more generally, of what we think and do. These conceptualizations have different implications when we apply them to WM. For instance, consider a situation in which WM receives an overload of information, some of which is relevant and some of which is irrelevant. Examples of this scenario are the complex-span paradigm (Daneman & Carpenter, 1980), in which to-be-remembered items alternate with stimuli to be processed but not retained, or the filtering paradigm (Vogel, McCollough, & Machizawa, 2005), in which participants see an array of visual stimuli and need to remember a pre-defined subset (e.g., only the red objects). According to theories assuming a limited resource allocated to representations in WM, attention limits how much of the given information can be retained, and a separate parameter determines the filtering efficiency, that is, the extent to which the cognitive system manages to keep the distractor information out of WM, so that it does not consume part of the valuable storage resource. These theories predict that individuals with lower WM capacity maintain a smaller amount of both relevant and irrelevant information, but their proportion, reflecting filtering efficiency, should be independent of WM capacity. According to the controlled-attention view, by contrast, the attentional resource determines the filtering efficiency. Hence, individuals with lower WM capacity retain the same amount of information as those with higher capacity, but people differing in WM capacity differ in the ratio of relevant to irrelevant information that they retain.

Paradoxes lurk when we try to combine the two notions of attentional resources, assuming that the same limited resource is required for both storage and control: According to this fusion version of the attentional-resource idea, keeping some irrelevant piece of information out of WM, or removing it from WM, consumes attentional resource (because it is an act of control over what we attend to) and at the same time frees up attentional resource (because it reduces the amount of information that is held in WM). In the same manner, stopping a cognitive process costs attentional resource but at the same time frees up attentional resource. With such a conception, it becomes virtually impossible to say whether some cognitive process – such as filtering or deleting information from WM – renders a net cost or a net gain in resource. As a consequence, the theory becomes untestable. This problem needs to be kept in mind when attempts are made to reconcile the two versions of attentional-resource theories of WM (e.g., Cowan, Fristoe, Elliott, Brunner, & Saults, 2006).^3^

If WM and the control of attention share a limited resource, we should expect substantial dual-task costs when an attention-control demand is combined with WM maintenance. Evidence for such a dual-task cost comes from studies demonstrating that a load on WM increases people’s susceptibility to distraction, for instance by the irrelevant stimuli in a flanker task (Kelley & Lavie, 2011; Lavie, Hirst, de Fockert, & Viding, 2004). Interpretation of this result is complicated by the observation that only a verbal WM load increases the flanker effect – a visual WM load has the opposite effect (Konstantinou, Beal, King, & Lavie, 2014; Konstantinou & Lavie, 2013). Konstantinou et al. (2014) explain this dissociation by assuming that visual WM contents place a load on a visual perceptual resource, and increasing the load on perceptual resources has been shown to reduce flanker interference (Lavie, 2005). In contrast, verbal WM relies on rehearsal for maintenance, and rehearsal competes for a shared attentional-control resource with the control of visual attention. The latter assumption is at odds with the position of most other resource theorists, who assume that rehearsal requires little, if any such resource (Baddeley, 1986; Camos, Lagner, & Barrouillet, 2009; Cowan, 2001). Other studies provide further evidence that a load on WM can both increase and decrease people’s distractability by a flanker stimulus during a perceptual comparison task: When the category of stimuli held in WM matched that of the targets of the comparison task (but not that of the flankers), the flanker compatibility effect increased, but when the WM contents matched the category of the flankers, and not the targets, then the flanker compatibility effect decreased under load compared to no load (Kim, Kim, & Chun, 2005; Park, Kim, & Chun, 2007). Taken together, there is no convincing evidence that loading WM depletes a resource needed for the control of attention.

We can also ask whether concurrent demands on the control of attention impair performance in a WM task. This appears not to be the case. The effect of concurrent processing on memory is larger when the processing task requires more attention control (e.g., task switching vs. task repetition, incongruent vs. neutral Stroop trials), but that effect is entirely accounted for by the longer duration of response selection in the more difficult conditions (Barrouillet, Portrat, & Camos, 2011; Liefooghe, Barrouillet, Vandierendonck, & Camos, 2008). Hence, the dual-task cost of concurrent processing for memory is a function of the demand on central attention for action selection, not the demand on the control of attention. Moreover, Lawrence, Myerson, Oonk, and Abrams (2001) found that when people had to make saccades to irrelevant locations during the retention interval, memory performance is impaired, in particular for spatial information. That effect was equally large for reflexive saccades towards a suddenly appearing target and for controlled anti-saccades away from a target, contrary to the assumption that the control of attention in the anti-saccade condition competes for WM resources. Bunting, Cowan, and Colflesh (2008) used a manual analog of the anti-saccade task as distractor activity during the retention interval, and found significantly worse performance in the anti-press than the pro-press condition in only 3 out of 12 experimental conditions.

A second prediction from the assumption that WM maintenance and controlled attention share a resource is that measures of the efficiency of the two should be correlated across individuals. This prediction has been tested with regard to two forms of control over the contents of WM (Hasher, Zacks, & May, 1999): *Filtering* irrelevant stimuli at encoding so that they never enter WM, and *removal* of no-longer relevant stimuli from WM after they have been encoded. Support for the prediction comes from studies measuring filtering efficiency in visual change-detection tasks through the effect of irrelevant stimuli on the CDA (Vogel et al., 2005). Individual differences in filtering efficiency are strongly correlated with accuracy in change detection (Luria et al., 2016). However, when Mall, Morey, Wolff, and Lehnert (2014) measured filtering efficiency through behavioral indicators – the performance gain from being able to ignore half the stimuli in the array, and the proportion of time people fixated on locations of irrelevant stimuli during encoding and retention – they found no correlation with people’s WM capacity, measured through complex-span tasks. One possible interpretation is that controlled attention (as indexed by filtering) and WM maintenance share a resource that is not domain general but rather specific to visual stimuli. Removal efficiency has been measured through the speed with which people remove to-be-updated information from WM in an updating paradigm (Ecker, Lewandowsky, & Oberauer, 2014). Whereas this first study showed no correlation of removal efficiency with WM capacity, a subsequent study measuring removal efficiency through a larger set of updating tasks observed a small positive correlation (Singh, Gignac, Brydges, & Ecker, 2018). This result could reflect a shared resource for WM maintenance and attentional control. Alternatively, it could mean that people who efficiently remove no-longer relevant information from WM are better at reducing interference from that information in WM, which improves their ability to retrieve the relevant information (Oberauer, Lewandowsky, Farrell, Jarrold, & Greaves, 2012).

Other research investigated the correlation between WM capacity and measures of attentional control outside the context of WM tasks, for instance the ability to attend to relevant and ignore irrelevant stimuli or features in perceptual decision tasks (e.g., the Stroop, flanker, or Simon task), the ability to suppress a strong action tendency (e.g., moving the eyes away from a suddenly appearing stimulus in the anti-saccade task), or the ability to stop an already prepared action (i.e., the stop-signal paradigm). Numerous studies have found positive correlations between WM capacity and these measures of attention control (e.g., Chuderski, 2014; McVay & Kane, 2012; Shipstead, Lindsey, Marshall, & Engle, 2014; Unsworth, 2015; Unsworth, Fukuda, Awh, & Vogel, 2014), whereas a few others failed to find such a relationship (Keye, Wilhelm, Oberauer, & van Ravenzwaaij, 2009; Wilhelm, Hildebrandt, & Oberauer, 2013). Additional support comes from findings of a positive correlation between WM capacity and people’s self-reported mind wandering in response to thought probes during a cognitive task (McVay & Kane, 2009, 2012; Randall, Oswald, & Beier, 2014).

Taken together, the evidence for a close relation between WM and the control of attention is mixed. The most convincing evidence comes from correlational studies linking WM capacity to indicators of attention control from tasks without a memory demand. There is some evidence that WM capacity is also correlated with the efficiency of controlling the contents of WM through filtering and removal, but it is yet too weak and inconsistent to draw strong conclusions. This correlational evidence, however, can be explained without invoking the notion of a shared resource, as I’ll discuss below (in the section “How is WM related to the control of attention and action?”). The experimental evidence from dual-task costs speaks against competition between WM maintenance and attention control for a shared resource.

I have considered three theoretical options for spelling out the idea of WM as relying on an attentional resource: (1) a shared resource for “storage” and “processing”, (2) a shared resource for perceptual attention and WM, and (3) a shared resource for attention control and WM. Of these three, the first option has received the most convincing empirical support, but it also suffers from empirical challenges, and from the conceptual problem of explaining how the competition for resources between storage and processing can have an impact on memory performance after the competition is over. I do not see these challenges as fatal – it is probably still too early to announce the “demise” (Klapp et al., 1983) of the idea that WM is limited by an attentional resource – but theorists working with this concept should aim to address these challenges. In the remainder of this article I discuss the relation of WM to attention from the perspective that attention is the selection and prioritization of information, which does not entail a commitment to a limited resource.

## Attention as Selection

A different perspective on the relation between WM and attention emerges when attention is defined not as a resource but as a mechanism for selecting and prioritizing representations. In this perspective, attention does not explain the capacity limit of WM. Rather, we should consider WM as an instance of attention – specifically, WM is attention to memory representations. Holding a set of representations in WM means selecting them from among all the representations that our mind is capable of, thereby rendering them available as input for cognitive operations. As such, WM meets the definition of attention as a mechanism of selection (Oberauer, 2009). In this perspective, the relationship between the concept of WM and the concept of attention is not an empirical but a conceptual one.

Nevertheless, we can ask several empirical questions about how WM is related to attention as a selection mechanism: (1) How is information selected into WM? (2) How is information selected within WM? (3) What is the relation between attention to memory and attention to perceived stimuli – are they the same, and if not, how do they influence each other? (4) How is WM related to the control of attention and action? I next address these questions in turn.

### How is Information Selected into Working Memory?

Information can be selected to be brought into WM from perception or from long-term memory. This selection is to a large extent controlled: People are very good, though not perfect, at letting only relevant information into WM. Moreover, people also have control over which information to keep in WM and which to remove.

**Filtering Perceptual Information.** With regard to perceived information, perceptual attention arguably plays an important role in selecting which stimuli are encoded into WM. Stimuli that are known to be irrelevant from the start, and are easy to discriminate from relevant stimuli, can be filtered out very effectively (Baddeley, Papagno, & Andrade, 1993), though not always perfectly (Ueno, Allen, Baddeley, Hitch, & Saito, 2011; Vogel et al., 2005); children and older adults seem to have more difficulty with filtering irrelevant stimuli at encoding (Sander, Werkle-Bergner, & Lindenberger, 2011). A question discussed in the context of visual WM is whether people can selectively encode relevant features but not irrelevant features of the same visual object. Some experiments show that relevant and irrelevant features of the same object have similar behavioral effects on memory performance (Marshall & Bays, 2013) and attentional capture (Gao et al., 2016; see the section on effects of WM on perceptual attention for an explanation of this effect). However, one fMRI study found that the relevant but not the irrelevant feature of a visual object could be reconstructed from the pattern of BOLD activity during the retention interval (Yu & Shim, 2017). Logie, Brockmole, and Jaswal (2011) have tested the effects of changes in irrelevant features on change-detection accuracy and found that such changes impair performance for retention intervals up to about 2 s but not thereafter. They propose that irrelevant features are initially encoded and subsequently removed from WM. This could explain why irrelevant features are not detectable in the sluggish BOLD signal that aggregates information over several seconds.

Filtering could be accomplished by perceptual selection – not attending to the irrelevant stimuli – but it could also be a separate selection step, such that a stimulus, even though selected for perceptual attention, is not encoded into WM. The latter possibility would imply that perceptual attention might be necessary, but is not sufficient for encoding them into WM. Evidence for this possibility comes from several sources. A series of experiments by H. Chen and Wyble (2015a, 2015b) used stimuli as attentional cues for a perceptual decision task, and after several trials inserted a surprise memory test for a feature of the cue. Although they have arguably attended to the cue because it was relevant for the decision task, people had poor memory for its features only a few seconds after its disappearance, suggesting that the stimulus, or at least the feature probed in the memory test, was not encoded into WM. When people expected the memory test, their performance was much better. In a related experiment H. Chen, Swan, and Wyble (2016) had participants visually track several moving target objects among distractors. To avoid confusing the targets with distractors participants had to continuously attend to them while they moved. Yet, in a surprise memory test they had little memory for the target’s colors.

A second source of evidence suggesting that attention is not sufficient to encode stimuli into WM comes from some of my experiments (Oberauer, 2018): Participants saw six words presented one by one in different screen locations; each word was followed by a cue to remember or forget it. The cue appeared only after word offset so that people had to attend to each word in case they would have to remember it. I also varied the time interval between each forget cue and the onset of the next word to manipulate how much time people had to remove a to-be-forgotten word from WM. The to-be-forgotten words had no effect on memory performance regardless of the cue-word interval, implying that they did not contribute at all to the load on WM.

These findings could mean that information, although attended, is not encoded into WM. Alternatively, the visual stimuli of Chen and Wyble, or the to-be-forgotten words in my experiments, could be encoded into WM but then removed very quickly so that their accessibility, and their effect on WM load, was not measurable even a few seconds later (see the section below on Removal). Perhaps neurophysiological markers of WM load with high temporal resolution, such as the CDA, could be leveraged to distinguish between these possibilities.

One limitation for efficient filtering (or removal) arises when people have to process the distracting material. When participants in my experiments (Oberauer, 2018) had to make a judgment on each word while it was on the screen, they could not entirely prevent encoding to-be-forgotten words into WM, though they were still able to diminish their effect on WM load relative to to-be-remembered words. Marshall and Bays (2013) found that comparing two stimuli during the retention interval of a visual WM task impaired WM performance as much as adding two more stimuli to the memory set, suggesting that encoding of these stimuli into WM could not be prevented at all.

**Selective Retrieval from Long-Term Memory.** Much of the information we process in WM comes from long-term memory. For the WM system to work effectively, it has to retrieve information from long-term memory selectively, so that only information useful for the current task enters WM (Oberauer, 2009). A demonstration of the effectiveness of this gating mechanism comes from experiments investigating the effect of previously acquired long-term memories on WM performance (Oberauer, Awh, & Sutterer, 2017). We had participants learn 120 associations between everyday objects and randomly selected colors. In a subsequent WM test they had to maintain three object-color conjunctions on each trial, and reproduce each object’s color by selecting it on a color wheel. Some of the objects in the WM test were objects for which they had learned an associated color before. These objects could reoccur in the WM test with their learned color – in which case retrieving the associated color should facilitate WM performance – whereas others reoccurred with a new random color – in which case retrieving the color from long-term memory should interfere with WM performance. We found evidence for proactive facilitation, but against proactive interference, implying that information from long-term memory is used if and only if the information in WM was so poor that drawing on long-term memory could only make things better.

**Removal of Information from WM.** The selection of which information to hold in WM is also controlled after encoding: Information no longer relevant must be rapidly removed so that it does not clutter WM (Hasher et al., 1999). There is a body of evidence showing that people can selectively remove no-longer relevant information from WM (for a review see Lewis-Peacock, Kessler, & Oberauer, 2018).

Removing an entire memory set when replacing it with a new one is a seamless and rapid process, though – as filtering – it is not perfect: Traces of the old memory set remain in WM, creating some mild proactive interference when items in the two sets are similar to each other (Ralph et al., 2011; Tehan & Humphreys, 1998), and a congruency benefit when the two sets partially overlap, sharing the same items in the same contexts (Oberauer, Souza, Druey, & Gade, 2013). Removal of a single item from the current memory set has been isolated experimentally as a process involved in WM updating (Ecker, Oberauer, & Lewandowsky, 2014). By contrast, removal is much less efficient when it comes to removing more than one item from a memory set but less than all of them: People find it difficult to remove a random subset of several items from a memory set. For instance, when informed, after encoding a list of six words, that the words in positions 2, 3, and 5 could be forgotten, there was no evidence that they did so – successful removal of a subset of three words was found only when they were already clearly marked as a separate subset at encoding (Oberauer, 2018). In sum, the efficiency of removal is limited by the ability to discriminate between to-be-maintained and to-be-removed contents of WM.

To conclude, the WM system is equipped with very efficient – though not perfect – mechanisms for controlling its contents through filtering perceptual input, selectively retrieving information from LTM, and removing no-longer relevant materials. Through these selection processes the cognitive system manages to usually have only the most relevant information for the current goal in WM.

### How is Information selected within WM?

Selecting information to be held in WM is a form of selection, but it not necessarily selection of one piece of information at the exclusion of all others: We often hold multiple separate items in WM simultaneously. Sometimes we have to select a single item from the set currently held in WM as the input for a process, or as the object of mental manipulation. Our ability to select individual items from the set currently held in WM points to a selection mechanism that I refer to as the *focus of attention* in WM (Oberauer, 2002; Oberauer & Hein, 2012). Evidence for the operation of such a narrow selection mechanism within WM comes from three observations: (1) In short-term recognition tests the last-presented item in a list is accessed at a faster rate than preceding items, and this has been interpreted as showing that the last-encoded item remains in the focus of attention (for a review McElree, 2006). (2) When an item in WM is needed as input for a cognitive operation (e.g., adding or subtracting a number from a particular digit in WM), or when one item needs to be selected as the object of an updating operation (e.g., replacing an item in WM by a new stimulus), then operating on the same WM item again in the next step takes less time than selecting another item from the memory set for the next operation. This item-switch cost (or item-repetition benefit) has been explained by assuming that the object of a cognitive operation remains in the focus of attention after the operation has been completed, and therefore does not need to be selected again when the same object is required for the next operation (Garavan, 1998; Oberauer, 2003). (3) After encoding a set of stimuli into WM, a retro-cue presented one to several seconds into the retention interval can guide attention to one item and thereby improve memory performance when that item is tested – often at the expense of performance when another item is tested (Griffin & Nobre, 2003; Landman, Spekreijse, & Lamme, 2003; for a review see Souza & Oberauer, 2016).

Whereas most of these empirical demonstrations come from situations in which a single item in WM needs to be selected, it has been argued that the focus of attention can hold more than one item (Gilchrist & Cowan, 2011). From the perspective of attention as selection, this should be feasible to the extent that selecting multiple items simultaneously does not undercut the purpose of selection. For instance, if the task is to update one out of several digits in WM through an arithmetic operation, selecting more than that one digit into the focus of attention would only lead to confusion – but if the task is to add two digits in WM together, selecting both of them into the focus of attention at the same time is arguably useful because then they could be used simultaneously as retrieval cues for the relevant arithmetic fact (Oberauer, 2013). Another situation in which it is functional to select two items into the focus simultaneously is when two tasks must be carried out simultaneously, one on each item, and the two items are sufficiently different to not risk cross-talk between the two tasks (Göthe, Oberauer, & Kliegl, 2016; Oberauer & Bialkova, 2011).

Using the retro-cue paradigm, neuroscience research has revealed a distinction between attended and unattended information in WM^4^: Whereas the attended information can be decoded from neural signals such as the pattern of BOLD activity over voxels, or the pattern of EEG activity over electrodes, the unattended information cannot – it remains neurally silent, but can be brought back into a neurally active state later by a retro-cue drawing attention to it (LaRocque, Lewis-Peacock, Drysdale, Oberauer, & Postle, 2013; Lewis-Peacock, Drysdale, Oberauer, & Postle, 2011; Sprague, Ester, & Serences, 2016) or by an uninformative strong input to the cortex (Rose et al., 2016; Wolff, Jochim, Akyürek, & Stokes, 2017). One recent study, however, paints a more differentiated picture: Decoding of orientations maintained in VWM from fMRI signals in visual cortex was again good for attended and absent for unattended items, but decoding from signals in parietal cortex (IPS and frontal eye fields) was equally good for both attended and unattended items – though much weaker than decoding of attended items in visual cortex (Christophel, Iamshchinina, Yan, Allefeld, & Haynes, 2018).

Behavioral evidence shows that retro-cues can be used to select not just individual items but also subsets of several items within WM (Oberauer, 2001, 2005), and selection of a subset can be followed by selection of an item within that subset (Oberauer, 2002). Therefore, we can distinguish three levels of selection in WM: (1) Selecting information to be in WM, constituting the current memory set, (2) selecting a subset of the memory set, and (3) selecting a single item from that subset. I have referred to these three levels as (1) the activated part of long-term memory, (2) the region of direct access, and (3) the focus of attention, respectively (see Oberauer, 2009, for a detailed discussion of the 3-level framework and evidence supporting it; and Oberauer et al., 2013, for a computational implementation). It is currently not clear whether more than one WM representation is neurally active (i.e., decodable from neural activity during the retention interval) at the same time, so we do not know whether the state of being neurally active characterizes the second or the third level of selection. One possibility is that during WM maintenance multiple representations – those in the direct-access region – are active at the same time, such that their pattern of neural activity is superimposed. Another possibility is that only one item – the one in the focus of attention – is neurally active at any time. If the focus of attention circulates among the items in WM, it would still be possible to decode several items from neural activation patterns (Emrich, Rigall, LaRocque, & Postle, 2013) because the temporal resolution of decoding from BOLD signals is lower than the speed at which the focus of attention shifts from one item to another (i.e., about 300 ms; Oberauer, 2003).

Univariate neural correlates of WM load, most notably the amplitude of the CDA (Vogel & Machizawa, 2004) and the BOLD activation in the inter-parietal sulcus (IPS) (Todd & Marois, 2004, 2005; Xu & Chun, 2006), imply that at least some form of persistent neural activity increases with the number of items maintained in WM. These neural measures, however, do not carry information about the content of WM, and therefore we do not know whether they reflect neurally active representations or some neural activity reflecting control processes that are involved in maintaining items selected. Another open question is whether these univariate measures of WM load reflect the first or the second level of selection – to find out we need studies that track these neural indicators of WM load while a retro-cue asks participants to select a subset of the current memory set: Does the neural marker track the set size of the subset or of the entire memory set? One study asking this question found that BOLD activation in IPS reflects the size of the entire memory set before the retro-cue but the size of the cued subset afterwards (Lepsien, Thornton, & Nobre, 2011), suggesting that IPS activation reflects the second level of selection, the direct-access region. In that study, however, participants were not asked to still maintain the not-cued subset in memory, so we don’t know whether they maintained it (at the third selection level, the activated part of LTM) or just removed it from WM.

A somewhat speculative hypothesis on how to reconcile all these findings is that univariate markers of WM load track the amount of information selected at the second level (i.e., the direct-access region). This information is maintained in WM through temporary bindings between contents and contexts through which they are accessible, probably in parietal cortex. These bindings are neurally silent – either because they are implemented through rapid synaptic plasticity (Mongillo, Barak, & Tsodyks, 2008) or because they are implemented in a pattern of neural activity that bears no similarity to the bound contents, such as a circular convolution of each content with its context (Eliasmith, 2013; Plate, 2003), so that they cannot be identified through decoding of the WM contents. However, neural activity patterns corresponding to the contents of the direct-access region could be re-activated during the retention interval by feeding non-specific activation into the contexts that act as retrieval cues for these contents, so that they could (faintly) be decoded from parietal cortical areas (Bettencourt & Xu, 2016; Christophel et al., 2018). This non-specific activation could be spontaneous noise in the neural network (Oberauer & Lin, 2017), or an attentional mechanism that selectively activates all contexts to which the contents of the direct-access region are bound. The content (or contents) selected for the third level of selection, the focus of attention, is represented in a neurally active fashion, probably in the prefrontal cortex (Bichot, Heard, DeGennaro, & Desimone, 2015; Mendoza-Halliday & Martinez-Trujillo, 2017), and this representation re-activates the corresponding sensory representation in those sensory cortical areas involved in its initial processing, so that the information in the focus of attention can be decoded from neural activity in those areas.

A prediction from this hypothesis is that when two to-be-remembered stimuli are presented sequentially, univariate markers such as the CDA should add up to reflect the combined load of both stimuli, whereas the decodability of the first stimulus should be substantially impaired by the encoding of the second, because the focus of attention abandons the first to encode the second stimulus. Evidence for the first assumption comes from studies showing that the CDA reflects the combined load of two successively presented parts of a memory set (Feldmann-Wüstefeld, Vogel, & Awh, 2018; Ikkai, McCollough, & Vogel, 2010); the second prediction remains to be tested.

### What is the Relation between WM and Perceptual Attention?

An extreme position would be that WM and perceptual attention are the same: By virtue of attending to a perceived stimulus, it is selected into WM. Maintaining stimuli in WM that are no longer present in the environment differs from perceptual attention only in the absence of the physical stimulus. The cognitive state is still the same, with the only difference that the representation in WM is arguably weaker and less precise due to the lack of informative sensory input. This extreme position is attractive due to its parsimony, but it is almost certainly wrong. We have already seen that perceptual attention to stimuli during the retention interval of a visual WM task leads to less interference than adding the same stimuli to WM (Fougnie & Marois, 2006). I have also discussed instances where stimuli were attended to and yet they leave hardly any trace in WM (H. Chen et al., 2016; H. Chen & Wyble, 2015a, 2015b; Oberauer, 2018). Moreover, single-cell recordings from monkey LPFC neurons showed partial but not complete overlap between the neurons responding selectively to a feature while it is perceptually attended and those doing so while the feature is being held in WM (Mendoza-Halliday & Martinez-Trujillo, 2017). If we accept that perceptual attention and WM are different entities, we can meaningfully ask how they causally affect each other.

**How does perceptual attention affect WM?** Some authors have argued that perceptual attention can be used to rehearse visual or spatial WM contents. The evidence for this idea is mixed. Some studies found a correlation between spontaneous eye movements during the retention interval – which presumably track visual attention – and recall success for sequences of spatial locations (Tremblay, Saint-Aubin, & Jalberg, 2006), but no such correlation was found for change detection in visual arrays (Williams, Pouget, Boucher, & Woodman, 2013). Directing people to attend to individual items in a visual array improves memory for those items relative to not-attended items in the array (Souza, Rerko, & Oberauer, 2015; Souza, Vergauwe, & Oberauer, 2018). However, it is not clear whether this effect relies on perceptual attention. Engaging perceptual attention by a secondary task during the retention interval (i.e., detection of a slight brightness change in the fixation cross) impaired performance in a visual change-detection task (Williams et al., 2013), but had at best a negligible effect on errors in a visual continuous-reproduction task, whereas engaging central attention impaired continuous reproduction more severely (Souza & Oberauer, 2017).

As discussed above in the section on Filtering, perceptual attention is probably necessary but not sufficient for encoding of stimuli into WM. Yet, filtering is not perfect, so that attended information is sometimes encoded into WM to some extent even when this is not desired. To the extent that this happens, we can expect that distractors presented during the retention interval of a WM task interfere with the to-be-remembered information, thereby impairing memory performance.

Evidence for such interference comes from studies of spatial WM. Van der Stigchel, Merten, Meeter, and Theeuwes (2007) found that recall of locations is biased towards the location of a suddenly appearing irrelevant stimulus on the screen, suggesting that this stimulus was inadvertently encoded into WM. Lawrence, Myerson, and Abrams (2004) had participants identify and compare two symbols during the retention interval of a WM task, which either appeared at fixation or in the periphery (left or right of fixation). When the symbols appeared in the periphery, spatial (but not verbal) WM performance was impaired more than for centrally displayed symbols. This suggests that attending to additional locations entails encoding these locations into WM to some degree, thereby interfering with memory for other locations. The interfering effect was stronger when participants were instructed to move their eyes to the peripheral symbols than when they were instructed to maintain fixation, in line with other findings showing that processing distractors enforces stronger encoding into WM than merely attending to them (Oberauer, 2018). Both studies unfortunately lack a control condition in which irrelevant stimuli are presented but not attended, so it is not clear how much perceptual attention contributes to their encoding into WM.

Does attending to a stimulus in the environment distract the focus of attention from information in WM? Two observations indicate that it might not: The beneficial effect of a retro-cue directing the focus of attention to one item in WM is not diminished by a subsequent task engaging perceptual attention (Hollingworth & Maxcey-Richard, 2013; Rerko, Souza, & Oberauer, 2014). Likewise, the object-repetition benefit in a spatial WM updating task was not diminished by requiring people to focus visual attention on a stimulus in the periphery in between updating steps (Hedge, Oberauer, & Leonards, 2015). However, the retro-cue effect probably arises in part from strengthening of the cued item’s binding to its context, and this effect lasts after the focus of attention has moved away from the cued item (Rerko et al., 2014; Souza et al., 2015). The same could be true for the object-repetition benefit: The item to be updated is selected into the focus of attention, and this strengthens the item’s binding to its context as a side effect, leaving that item temporarily more accessible than other items even if the focus of attention moves away from it. Evidence suggesting that attending to perceptual stimuli does distract the focus of attention comes from studies using multivariate neural signals to read out the information in the pattern of neural activity. The decodability of a single item in WM is drastically diminished – at least temporarily – by the onset of an irrelevant stimulus, or just by the person attending to a location in anticipation of a stimulus, during the retention interval (Bettencourt & Xu, 2016; van Moorselaar et al., 2017). However, in these studies the irrelevant stimulus hardly affected memory performance. Therefore, an alternative possibility is that the content of the focus of attention is represented in pre-frontal cortex (Bichot et al., 2015), and the corresponding sensory representations are merely epiphenomenal, so that the elimination of the latter does not imply a distraction of the focus of attention in WM.

To conclude, surprisingly little can be said with confidence: Perceptual attention to stimuli often – but not always – leads to them being encoded into WM to some extent, so that they interfere with similar information. The use of perceptual attention for rehearsal has not been demonstrated convincingly. Whether the focus of attention can stay on an item in WM while perceptual attention engages with a different stimulus in the environment is still unclear.

**How does information in WM affect perceptual attention?** It appears plausible that holding some information in WM tends to draw perceptual attention to similar information in the environment, thereby facilitating its processing. Initial evidence for that assumption comes from experiments by Awh et al. (1998): Holding the spatial location of an object in WM facilitates processing of other stimuli appearing in the same location during the retention interval. A subsequent similar study taking additional measures to discourage eye movements, however, failed to replicate this finding (Belopolsky & Theeuwes, 2009).

A more specific version of the same idea is the assumption that the item held in the focus of attention in WM – usually a single item – functions as a “search template”, guiding perceptual attention to matching stimuli (Olivers, Peters, Houtkamp, & Roelfsema, 2011). This idea has received considerable empirical support from studies of the “attentional capture” effect in visual search: When people are asked to hold an item in WM – for instance a color, or just a color word – and carry out a visual search task during the retention interval, attention is drawn to stimuli in the search display matching the item in WM (Soto, Hodsoll, Rotshtein, & Humphreys, 2008). When more than one item is held in WM and one of them is retro-cued, then only the retro-cued item causes attentional capture (Mallett & Lewis-Peacock, 2018; van Moorselaar, Battistoni, Theeuwes, & Olivers, 2014; van Moorselaar, Theeuwes, & Olivers, 2014). This finding provides further evidence for the special functional status of representations in the focus of attention (i.e., the third level of selection).

### How is WM related to the control of attention and action?

Some theorists argue for a close relation of WM specifically to controlled attention (Kane et al., 2001; McVay & Kane, 2009; Unsworth et al., 2014). The evidence for this link comes primarily from correlations between measures of WM capacity and controlled attention (reviewed above in the section on resources for attention control). There are at least two interpretations of this correlation. One is that people with high ability to control their attention are good at keeping irrelevant contents out of WM (Hasher & Zacks, 1988), either by filtering them out at encoding (Vogel et al., 2005) or by removing them once they are no longer relevant (Oberauer et al., 2012), and therefore they make better use of their WM capacity. This account has difficulties explaining why measures of controlled attention were found to correlate substantially also with measures of (visual) WM in which no irrelevant stimuli were presented, and no contents need to be removed from WM (Unsworth et al., 2014).

A second explanation, which I believe to be more promising, implies the reverse direction of causality. It starts from the assumption that the main function of WM is to hold representations that control what we think and do, including what we direct our attention to (Oberauer, 2009). For instance, in visual search perceptual attention can be controlled by holding a template of the search target in the focus of attention in WM (Olivers et al., 2011). Selection of responses to stimuli in accordance with the currently relevant task goal is accomplished by holding a task set – a representation of the relevant stimulus categories, the response options, and the mapping between them – in WM (Monsell, 2003; Oberauer et al., 2013). In both cases, control could also rely on representations in long-term memory. For the case of visual search, Woodman, Carlisle, and Reinhart (2013) present strong evidence that search targets that repeat across successive trials are held in WM only for the first few trials, after which search is controlled by target representations in long-term memory. The finding that search becomes more efficient with practice when the same set of stimuli is consistently used as targets or distractors further underscores the role of long-term memory in controlling perceptual attention in search tasks (Shiffrin & Schneider, 1977). For the case of response selection, practicing a task with consistent stimulus-response mappings leads to long-term learning of these mappings, greatly improving task performance. Representations in WM are necessary for control when we want to do something new – searching for a new target, or carrying out a new task that we just learned from instruction. WM representations are particularly important when the new action is inconsistent with one that we have learned – for instance, searching for a target that used to consistently figure as distractor, or switching from one task to another that maps the same stimuli to new responses. In these cases, WM provides a medium for building and maintaining new representations that control our cognitive processes and actions, if necessary countermanding our long-term knowledge. On these assumptions, the correlation between WM capacity and performance in controlled-attention tasks arises because people with better WM capacity have better (i.e., more robust, more precise) representations in WM of the (cognitive or overt) action they intend to carry out, such as search templates and task sets.

To conclude, I argue that WM plays a crucial role in controlling attention and action by holding the representations that guide attention and action. The control process consists of selecting these representations into WM – once they are established in WM, they have their influence on attention and action automatically: Perceptual attention is “captured” by stimuli matching the content of the focus of attention even when this is only detrimental to performance in the current task (Foerster & Schneider, 2018; Gao et al., 2016); newly instructed tasks, once implemented as task sets in WM, function like a “prepared reflex”, influencing response selection even when they are currently not relevant (Meiran, Liefooghe, & De Houwer, 2017).

## Conclusions

Attention is closely related to WM. Unpacking this relationship reveals many different ways in which the WM-attention link can be spelled out. A first divide is between theoretical ideas about attention as a resource on the one hand, and about attention as a mechanism for selecting and prioritizing information on the other. The first approach entails the theoretical commitment that a limited attentional resource is at least in part responsible for the capacity limit of WM. This assumption has considerable empirical support but also significant weaknesses (for a review see Oberauer et al., 2016), so that researchers should not endorse it as a default. The second approach does not imply a commitment to any assumptions about WM or attention, and therefore offers a more neutral starting point for asking how the two are related. From the theoretical considerations and the evidence reviewed here I conclude that the following assertions about specific relations between attention and WM are justified:

1. By virtue of holding a selected subset of all available representations in memory, WM is by definition a form of attention.
2. The selection of information to be held in WM is a form of controlled attention: The selection of stimuli to be encoded into WM is controlled by a filtering mechanism set according to our intentions; the retrieval of long-term memory information into WM is gated to admit only information relevant for our current goals, and information no longer relevant for our current goal is removed from WM.
3. Attending to a perceived stimulus probably facilitates encoding of that stimulus into WM, but does not mandate it. Even attended information can be, to a large extent, filtered out.
4. Within the contents of WM the focus of attention can be directed to individual items, or subsets of items, selected for manipulating them, or as input for processes (e.g., mental arithmetic, visual search).
5. Control of attention and action relies on representations in WM that guide attention and action, such as search templates and task sets, especially when these are new and in conflict with knowledge in long-term memory. Once established in WM, these representations control attention and action independently of our intentions.

Unsurprisingly, there are also many things we don’t know. Table 2 presents a non-exhaustive list of open questions that I believe future research should address with high priority. I hope that this effort will lead to an increasingly more precise and nuanced picture of how WM is related to attention.

**Table 2:** Open Questions.

Topic	Question

Relation of central attention to WM	Under which circumstances – in particular, for how long into the retention interval – does an attention-demanding processing task compete with maintenance in WM?
Relation of perceptual attention and WM	Is the capacity limit of perceptual attention caused by the same limiting factors as the capacity limit of WM?
	To what extent does perceptual attention to a stimulus lead to its encoding into WM even without the intention to encode it?
The focus of attention in WM	Is the focus of attention in WM the same as the focus of perceptual attention, so that directing attention to a perceived stimulus diverts the focus from its current content in WM, and vice versa?
	Is the distinction between WM contents in and outside of the focus of attention a qualitative difference or merely a quantitative difference (in degree of memory strength or activation)?
	How many distinct items can be selected simultaneously into the focus of attention so that they guide perceptual attention? Some have argued that it is only one item at a time (van Moorselaar, Theeuwes, et al., 2014); others argue for more than one (Hollingworth & Beck, 2016)
The role of neurally active representations	Are all contents of WM represented in a neurally active manner that allows decoding of their contents from neural signals, or only a selected subset of WM contents – maybe only a single item at a time?
	Are neurally active representations in sensory cortex functionally important for maintenance in WM, or merely an epiphenomenon arising from back-projection of WM representations into sensory areas?
Relation between WM and the control of attention	Under which conditions does a concurrent load on WM impair the control of attention in conflict tasks (e.g., flanker, Stroop tasks)?
	What causal relation underlies the correlation between WM capacity and measures of attention control (e.g., filtering in visual WM tasks; anti-saccade performance, mind wandering)?

## Footnotes

I will use the term *object* (of attention) in a broad sense, referring to every entity that we can pay attention to (e.g., physical objects, events, people, concepts and ideas, goals and actions, …).

Chun et al. (2011) refer to this distinction as “internal” vs. “external” attention. I find this terminology misleading: The memory of a tree is not more internal than the perception of a tree: Both are internal representations of external objects.

Another paradoxical implication of the fusion account is that, once the resource is completely absorbed for storage purposes, there is no resource left for control processes clearing irrelevant material from WM, and once an ongoing process monopolizes the entire attentional resource, there is no way of stopping it. A meta-control process is necessary to ensure that there is always enough resource left for control processes. If the meta-control process needs a share of the resource for itself, we are on the way to an infinite regress.

The term “unattended” is to be understood relative to the “attended” content of WM. At the same time, all contents of WM are prioritized over all other memory representations, and as such are attended, though on a broader level of selection.
